# Diffusion Tensor Imaging-Based Glymphatic Dysfunction Assessments in Migraine Syndromes: Mechanisms and Diagnostic Implications

**DOI:** 10.3390/biomedicines13122981

**Published:** 2025-12-04

**Authors:** Emily Lai, Joshua Estin, Jiahao Zhou, Roger Sheffmaker, Manisha Koneru

**Affiliations:** 1 Department of Neurosciences, Cooper Medical School of Rowan University, Camden, NJ 08103, USA; laiemi87@rowan.edu (E.L.); estinj96@rowan.edu (J.E.); zhouji84@rowan.edu (J.Z.); sheffm17@rowan.edu (R.S.); 2 Cooper Neurological Institute, Cooper University Health Care, Camden, NJ 08103, USA

**Keywords:** diffusion tensor imaging, headache, magnetic resonance imaging, migraine disorders, neuroimaging

## Abstract

Migraine is a common neurological disorder. Impaired glymphatic clearance has been recently implicated in the pathogenesis of migraine. Diffusion tensor imaging (DTI) metrics have been explored as a tool for assessing glymphatic status. The objective is to summarize recent advances in identifying potentially useful DTI metrics in migraine patient populations. Since 2020, there has been mixed evidence regarding the applicability of various DTI metrics in migraine subpopulations. Most studies focused on whole-brain analyses, or specified regions of interest along the perivascular space, to extract quantitative parameters; most studies compared differences in these parameters associated with a migraine diagnosis, or were aiming to assess correlation between these parameters and migraine subtypes. Thus, early studies have demonstrated conflicting results regarding the utility and applicability of DTI for migraine. Greater insight into the molecular basis between migraine pathophysiology and the glymphatic system might help shape approaches to analyzing DTI data for migraine patients. Future studies incorporating larger cohorts and integrating advanced data analytics may provide additional information for the role of DTI in migraine.

## 1. Introduction

Migraine is a widespread neurological disorder that affects more than one billion people worldwide, particularly women under the age of 50 [1]. Despite extensive research, the pathophysiology remains incompletely understood. The complexity of migraine diagnosis and pathogenesis necessitates the exploration of novel therapeutic targets. Currently, three general mechanisms are thought to underlie migraine and aura development: neuroinflammation, excess calcitonin gene-related peptide (CGRP), and cortical spreading depression [2].

The glymphatic system, responsible for waste clearance in the central nervous system, has emerged as a potential contributor to migraine pathogenesis. This system facilitates cerebrospinal fluid (CSF) and interstitial fluid exchange, thereby clearing neurotoxic metabolites. In this way, dysfunction of the glymphatic system could alter the three aforementioned mechanisms underlying migraine. Recently, the emergence of diffusion tensor imaging (DTI) for assessing glymphatic activity has allowed researchers to examine its role in migraine patients, offering new insights into this potential process. In this review, we aim to summarize the current proposed mechanism of glymphatic dysfunction in migraine pathogenesis, describe recent applications of DTI to quantify glymphatic dysfunction to assess migraine phenotype, and underscore limitations of current studies to guide future directions.

## 2. Glymphatic Dysfunction in Migraine

The glymphatic system plays a critical role in maintaining brain homeostasis by clearing harmful solutes from the CSF. The exchange between the CSF and interstitial fluid is accomplished through aquaporin-4 (AQP4) channels on astrocyte endfeet [3]. In the context of migraine, cortical spreading depressions are the neural events that underlie aura. It has been shown that during cortical spreading depressions, the paravascular space transiently collapses, impairing glymphatic flow [4]. This disruption results in the accumulation of key substances such as potassium, glutamate, and CGRP [5]. CGRP is specifically of interest, as it is an important mediator of headaches, particularly migraine. During migraine attacks, CGRP, a vascular neuropeptide, is released peripherally at perivascular trigeminal afferents and cannot cross the blood–brain barrier [6]. Despite this, CGRP concentration is five times higher in CSF compared to plasma after activating dural afferents, showing that CGRP may penetrate the CSF via the glymphatic system [7]. CGRP is essential for the nociceptive transmission of migraine and has been shown to be persistently and excessively released during migraine; it induces the vasodilation of meninges and intracranial arteries as well as precipitating a local inflammatory response, causing the symptoms of a migraine. Impaired glymphatic clearance of CGRP may therefore cause neuronal hyperexcitability and exacerbate migraine duration and severity [8]. 

Furthermore, glymphatic dysfunction may contribute to migraine pathology by impairing the removal of reactive oxygen species and pro-inflammatory cytokines [2]. Studies in animal models have demonstrated that pharmacological inhibition of AQP4 worsens migraine symptoms, seen through worsened hyperalgesia [9]. This highlights the critical role of AQP4 in maintaining glymphatic function in migraine [10,11,12]. These findings highlight the need for further investigation into the glymphatic system’s role in migraine pathogenesis and its potential as a therapeutic target.

## 3. Diffusion Tensor Imaging and Glymphatics

Glymphatic function may be assessed with DTI to evaluate fluid dynamics and metabolite clearance in the brain. DTI-ALPS (DTI analysis along the perivascular space) is a non-invasive magnetic resonance imaging (MRI) technique used to evaluate glymphatic system activity. In the MRI acquisition process, the DTI-ALPS method can be added to the standard MRI sequences, typically requiring an additional 5–10 min to incorporate dedicated diffusion-weighted imaging protocols to gather metrics regarding water diffusion along the perivascular spaces (PVS). The DTI-ALPS method calculates the ALPS index, a ratio comparing water diffusivities along different axes in the lateral ventricle body plane along the perivascular space. Specifically, it compares diffusion along two key axes: the direction parallel to the perivascular spaces, typically aligned with white matter tracts, and the direction perpendicular to these spaces [13]. Higher ALPS index values correspond to more effective glymphatic function, indicative of efficient cerebrospinal fluid and interstitial fluid exchange, as well as waste clearance along the perivascular pathways [14]. This method assesses the structural and fluid dynamics supporting glymphatic clearance, providing an indirect measure of glymphatic function. 

Studies have shown that a significantly lower ALPS index is seen in patients with various forms of cognitive impairment and neurological conditions such as idiopathic normal pressure hydrocephalus and traumatic brain injury. For example, in a study of normal pressure hydrocephalus patients, the ALPS index was significantly lower compared to healthy controls. This lower ALPS index was associated with disease severity and cognitive impairment, as measured by motor function tests such as the Timed Up and Go test and cognitive assessments such as the Mini-Mental State Examination [15]. Similarly, in patients with Alzheimer’s disease, mild cognitive impairment, and vascular cognitive impairment, ALPS index was significantly lower compared to normal controls. Liang et al. found a positive correlation between the ALPS index and cognitive scores, suggesting that the lower water diffusivity of an impaired glymphatic system along the perivascular spaces is associated with cognitive decline in dementia patients [16]. 

In post-processing of DTI data, metrics such as fractional anisotropy (FA), mean diffusivity (MD), axial diffusivity (AD), and radial diffusivity (RD) are extracted to assess white matter integrity. Water diffusion in the brain is largely restricted by neuronal fiber tracts, and FA values reflecting the directionality of water diffusion provide insight into the microstructural organization and integrity of white matter tracts and PVS [17]. A high FA indicates well-organized, healthy white matter, and a low FA suggests disrupted white matter. FA values range from 0 (isotropic diffusion), reflective of equal water diffusion in all directions and disrupted or absent microstructural barriers, to high anisotropy and FA values up to 1 (infinite anisotropic diffusion), in which water diffusion is strongly directional, reflecting well-organized and intact axonal bundles, myelin sheaths, or well-organized PVS. MD serves as a measure of the overall magnitude of water diffusion, regardless of directionality. AD represents diffusion along the fiber tracts, parallel to axons, while RD represents diffusion perpendicular to the fiber tracts, alongside the myelin sheaths. High MD is suggestive of higher diffusion, often due to tissue damage and disintegration, vasogenic edema, interstitial fluid accumulation, or neurodegeneration. High AD may indicate axonal damage, and high RD suggests demyelination. These imaging biomarkers provide critical insights into conditions associated with tissue integrity and water diffusion, offering valuable diagnostic and prognostic information for a variety of neurological conditions.

## 4. Advances in Diffusion Tensor Imaging for Migraine

Clinical imaging studies with DTI have revealed mixed results. In total, 22 studies published between 2020 and 2024 analyzed migraine patients with DTI (Table 1). The most common metrics of interest included DTI-ALPS, FA, MD, RD, and AD.

### 4.1. DTI-ALPS

Five studies examined DTI-ALPS as the metric of interest [8,27,34,36,38]. There were no significant differences when comparing episodic migraine patients to healthy controls [8,27,38]. Additionally, there were no DTI-ALPS differences between patients with versus without aura, or based on presence of radiographic white matter hyperintensities [27,34]. Two studies performed a three-way comparison between episodic migraine patients, chronic migraine patients, and healthy controls. However, Zhang et al. (2023) observed increased right-sided DTI-ALPS in chronic migraine patients compared to the other groups, whereas Wu et al. (2024) found decreased DTI-ALPS in chronic migraine patients [8,38]. Based on the literature, there appear to be no differences in DTI-ALPS between episodic migraine patients and matched controls, whereas comparing DTI-ALPS in chronic migraine patients with matched controls has so far yielded contradictory results.

### 4.2. Additional DTI Metrics

Of the remaining seventeen studies, two studies examined FA and apparent diffusion coefficient (ADC) [26,35]; four studies examined FA and MD [21,25,32,37]; eleven studies examined FA, MD, RD, and AD [18,19,20,22,23,24,28,29,30,31,33]. Structures where DTI differences were repeatedly observed include the corpus callosum, thalamus, and internal capsule.

Reduced FA has been detected in the corpus callosum of migraine patients compared to healthy controls [26,37]. Coppola et al. (2020) detected lower FA in chronic migraine patients than in episodic migraine patients, but did not find any significant differences when each migraine group was compared to healthy controls [18]. One subgroup of episodic migraine patients with reduced FA compared to controls appears to be episodic migraine patients with cutaneous allodynia [22]. Between chronic and episodic migraineurs, chronic migraineurs have reduced AD in the corpus callosum [20]; both reduced MD and increased MD have been reported [18,33]. While reduced FA was noted in the thalamus of migraine patients compared to healthy controls, no differences were observed when restricting the patient sample to episodic migraineurs, implying this effect may be driven by chronic migraineurs [21,25,30,37]. While the internal capsule appears to have reduced FA in migraine patients overall, this finding was not observed in a subset of chronic migraineurs or episodic migraineurs [18,20,25,33,37].

When comparing migraine patients to matched controls, both increased MD and decreased MD have been observed within the internal capsule [25,33,37]. Contradictory DTI findings between different studies have also been observed at the cerebellar white matter tracts and the periaqueductal gray [20,25,28,29,32,37]. Similarly, multiple studies performing whole-brain DTI analysis found no significant differences in any white matter tracts when comparing episodic migraineurs to matched controls [18,20,23,33].

In summary, review of the recent literature examining DTI changes in migraineurs relative to matched controls yielded a wide range of occasionally contradictory findings. This inconsistency may be due to heterogeneity of disease presentation, differences in study inclusion and exclusion criteria, or unknown covariates [39,40].

## 5. Future Directions

Available evidence suggests that DTI may be a valuable diagnostic and prognostic tool for migraine, but data are contradictory. Future studies may want to include larger cohorts to validate DTI as a diagnostic or prognostic tool for migraine. A larger sample size will account for diverse patient populations, variability in migraine presentation and prognosis, and resolve the current inconsistencies in existing findings. Additionally, ongoing prospective studies may elucidate the uses of DTI as a tool for migraine. In the ongoing “International Headache Registry Study” (NCT05418218), the researchers plan to conduct a prospective cohort over 60 years, collecting data from migraine patients aged 4–99 [41]. The DTI metrics collected in this study might clarify confusion surrounding migraine prognosis. Another study, “Study of the Glymphatic System in Migraine” (NCT05907655), aims to enroll 50 patients to further analyze the role of the glymphatic system in migraine attack initiation [42]. The researchers intend to induce migraine via nitroglycerine administration and assess the relationship between the nitroglycerine-induced migraine attacks and glymphatic dysfunction. These studies exemplify the potential of DTI uses in advancing understanding of migraine mechanisms and improving diagnostic capabilities.

Greater insight into migraine pathophysiology and the potential role of the glymphatic system might enhance the clinical utility of DTI in migraine patients. With such insights, DTI may be a valuable tool for earlier and more precise diagnoses. Also, DTI metrics may allow clinicians to quantify migraine treatment effectiveness or monitor migraine attack progression. In addition to nitroglycerin-induced migraine models, additional migraine models, such as CGRP-induced and potassium channel modulators (i.e., levcromakalim)-induced models, may be developed to study pathogenesis and correlate phenotype with biochemical and radiographic features [43]. A deeper understanding into the pathophysiology of migraine attacks may also uncover new potential therapeutic targets.

Technological advancements, such as artificial intelligence and radiomics-based feature extraction hold promise for refining DTI data analytics. Deep learning approaches enabling automated segmentation and analysis of explainable anatomical variants, including those in the perivascular space beyond DTI-ALPS, may offer additional accuracy in identifying clinically significant diagnostic and therapeutic data. Similarly, integrating advanced modeling techniques, such as with machine learning approaches over traditional multivariable modeling approaches, may augment the yield from these endeavors, as it expands the ability to introduce multiple data points from a wide range of domains, inclusive of multiple imaging sequences and clinical data. These methods may detect subtle and previously undetected biomarkers; enhanced processing may enable integration of multiple magnetic resonance imaging modalities in addition to DTI, allowing more robust characterization of imaging features and pathophysiological correlates. Overall, future studies should incorporate larger cohorts, explore innovative applications of DTI, and integrate advanced data analytics to deepen our understanding of migraine.

## 6. Conclusions

DTI is a noninvasive imaging modality used to assess glymphatic system activity and its potential association with migraine. While promising, current studies present conflicting findings regarding DTI metrics in migraine patients. Further research is essential to establish standardized DTI protocols and clarify its diagnostic and clinical applications in migraine. Investigation of translational applications of DTI could improve diagnostic precision, enable treatment monitoring, and identify new therapeutic targets, ultimately enhancing patient care and clinical outcomes.

## Figures and Tables

**Table 1:** Overview of diffusion tensor imaging findings in migraine.

Study	Patient Numbers	Migraine Subtype	DTI Metrics	Regions of Interest	Summary of Findings
Coppola et al., 2020 [18]	19 EM, 18 CM, 18 HCs	EM, CM	FA, MD, RD, AD	Whole brain	EM vs. HC: no differences CM vs. HC: ↑ RD, ↑ MD in multiple areas CM vs. EM: ↓ FA, ↑ MD in multiple areas
Mungoven et al., 2020 [19]	39 EM, 39 HCs	EM with and without aura	FA, MD, RD, AD	Trigeminal nerve within pontine cistern	EM vs. HC: ↓ FA at left trigeminal nerve root entry zone; this effect was driven by differences in the middle ⅓ and rostral ⅓ of CN 5 ↑ RD in left middle ⅓ and rostral ⅓, but no significant differences when comparing the whole root entry zone
Planchuelo-Gómez et al., 2020 [20]	54 EM, 56 CM, 50 HCs	EM, CM	FA, MD, RD, AD	Whole brain	Uncorrected for duration of migraine history: No significant differences in EM vs. HCs, CM vs. HCs CM vs. EM: ↓ AD in 38 regions Corrected for duration of migraine history: CM vs. EM: ↓ AD in middle cerebellar peduncle only EM vs. HCs: ↑ AD in 7 left-sided areas All results were unchanged between including vs. excluding patients with aura
Porcaro et al., 2020 [21]	20 EM w/o aura, 20 HCs	EM without aura	FA, MD	Thalamus	No significant differences
Russo et al., 2020 [22]	47 EM, 19 HCs	EM without aura	FA, MD, RD, AD	Whole brain	EM with cutaneous allodynia vs. HCs: ↓ FA in corpus callosum EM_with CA_ vs. EM_without CA_: ↓ FA in corpus callosum
Masson et al., 2021 [23]	19 EM, 19 HCs	EM without aura	FA, MD, RD, AD	Whole brain	No significant differences
Porcaro et al., 2021 [24]	20 migraine w/o aura, 20 HCs	EM without aura	FA, MD, AD, RD	Hypothalamus	EM vs. HCs: ↑ MD, ↑ AD, ↑ RD in hypothalamus Exploratory analysis: ↓ FA in L/R posterior hypothalamus. ↑ MD, ↑ AD, ↑ RD in anterior and posterior hypothalamus bilaterally
Taman et al., 2021 [25]	33 migraine, 15 controls	Migraine without aura	FA, MD	Gray and white matter	Migraine vs. controls: ↓ FA, ↑ MD in R thalamus, R globus pallidus, R/L hippocampus head ↓ FA in R/L frontal lobe white matter, R/L posterior internal capsule, R/L cerebellar white matter ↑ MD in R/L frontal lobe white matter, R posterior internal capsule, R/L cerebellar white matter
Tantik Pak et al., 2021 [26]	25 migraine with MOH, 33 migraine without MOH	Migraine with vs. without medication overuse headache	FA, ADC	Orbitofrontal cortex	Migraine with MOH vs. migraine without MOH: ↑ FA in left orbitofrontal cortex
Lee et al., 2022 [27]	92 EM, 80 HCs	EM with and without aura	DTI-ALPS	Along the perivascular space	EM vs. HCs: no significant ALPS difference EM with aura vs. EM without aura: no significant ALPS difference
Mungoven et al., 2022 [28]	38 EM, 38 HCs	EM with and without aura	FA, MD, RD, AD	Whole brain	EM vs. HCs: ↑ MD, ↑ AD, ↑ RD in spinal trigeminal nucleus, periaqueductal gray, primary visual cortex
Shi et al., 2022 [29]	60 CM, 60 HCs	CM with and without aura	FA, MD, AD, RD	Autonomic-related brain regions	CM vs. HCs: no significant differences in autonomic-related brain regions
Yang et al., 2022 [30]	27 EM, 30 HCs	EM with and without aura	FA, MD, RD, AD	Thalamus	EM vs. HCs: no significant differences
Abagnale et al., 2023 [31]	20 migraine with pure visual aura, 15 migraine with complex aura, 19 HCs	Migraine with pure visual aura vs. migraine with complex neurological auras	FA, MD, AD, RD	Whole brain	Migraine with visual aura vs. migraine with complex aura vs. HCs: no significant differences
Dobos et al., 2023 [32]	37 EM, 40 HCs	EM without aura	FA, MD	Whole brain	EM vs. HCs: ↑ FA in 13 regions ↓ FA in 5 regions ↓ MD in L cerebellum crus, pons
Martín-Martín et al., 2023 [33]	51 EM, 56 CM, 50 HCs	EM, CM	FA, MD, AD, RD	Whole brain	CM vs. EM: ↓ MD in 38 regions ↓ AD in 40 regions CM vs. HC: no significant differences EM vs. HC: no significant differences
Ornello et al., 2023 [34]	147 migraine patients, no controls	Migraine with or without WMH	DTI-ALPS	Along the perivascular space	Migraine with WMH versus without WMH: no significant ALPS difference
Tantik Pak et al., 2023 [35]	51 migraine, 44 HCs	Migraine with or without aura, with or without MOH	FA, ADC	Corpus callosum	Migraine vs. HCs: ↓ FA in corpus callosum genu
Zhang et al., 2023 [8]	32 EM, 24 CM, 41 HCs	EM, CM	DTI-ALPS	Along the perivascular space	CM vs. EM, CM vs. HCs: ↑ right-sided DTI-ALPS No group differences in left-sided DTI-ALPS
Cao et al., 2024 [36]	37 migraine, 29 HCs	N/A	DTI-ALPS and DKI-ALPS	Along the perivascular space	No significant differences in DTI-ALPS Migraine vs. HCs: ↑ right DKI-ALPS
Nanda and Sachdev, 2024 [37]	30 migraine, 20 HCs	CM and EM, with and without aura	MD, FA	White matter tracts	Migraine vs. HCs: ↓ FA in 5 regions ↓ MD in 5 regions
Wu et al., 2024 [38]	112 migraine, 63 HCs	EM, CM with and without MOH	DTI-ALPS	Along the perivascular space	CM vs. EM, CM vs. HCs: ↓ ALPS EM vs. HCs: no significant differences CM with MOH vs. CM without MOH: ↓ ALPS

Abbreviations: ↑, increased; ↓, decreased; EM, episodic migraine; CM, chronic migraine; HCs, healthy controls; FA, fractional anisotropy; MD, mean diffusivity; RD, radial diffusivity; AD, axial diffusivity; ADC, apparent diffusion coefficient; DTI-ALPS, diffusion tensor imaging along the perivascular space; DKI-ALPS, diffusion kurtosis imaging along the perivascular space; R, right; L, left; CA, cutaneous allodynia; MOH, medication overuse headache; WMH, white matter hyperintensities.

## Data Availability

No new data were created or analyzed in this study. Data sharing is not applicable to this article.

## Abbreviations

The following abbreviations are used in this manuscript:

ADC	Apparent diffusion coefficient
AQP4	Aquaporin-4
AD	Axial diffusivity
CGRP	Calcitonin gene-related peptide
CSF	Cerebrospinal fluid
DTI	Diffusion tensor imaging
DTI-ALPS	Diffusion tensor imaging along the perivascular space
FA	Fractional anisotropy
MRI	Magnetic resonance imaging
MD	Mean diffusivity
PVS	Perivascular space
RD	Radial diffusivity
